# High Incidence of Unplanned Pregnancy after Antiretroviral Therapy Initiation: Findings from a Prospective Cohort Study in South Africa

**DOI:** 10.1371/journal.pone.0036039

**Published:** 2012-04-27

**Authors:** Sheree R. Schwartz, Helen Rees, Shruti Mehta, Willem Daniel Francois Venter, Taha E. Taha, Vivian Black

**Affiliations:** 1 Wits Reproductive Health and HIV Institute, University of the Witwatersrand, Johannesburg, South Africa; 2 Department of Epidemiology, Johns Hopkins Bloomberg School of Public Health, Baltimore, Maryland, United States of America; London School of Hygiene and Tropical Medicine, United Kingdom

## Abstract

**Background:**

Increased fertility rates in HIV-infected women receiving antiretroviral therapy (ART) have been attributed to improved immunological function; it is unknown to what extent the rise in pregnancy rates is due to unintended pregnancies.

**Methods:**

Non-pregnant women ages 18–35 from four public-sector ART clinics in Johannesburg, South Africa, were enrolled into a prospective cohort and followed from August 2009–March 2011. Fertility intentions, contraception and pregnancy status were measured longitudinally at participants' routine ART clinic visits.

**Findings:**

Of the 850 women enrolled, 822 (97%) had at least one follow-up visit and contributed 745.2 person-years (PY) at-risk for incident pregnancy. Overall, 170 pregnancies were detected in 161 women (incidence rate [IR]: 21.6/100 PY [95% confidence interval (CI): 18.5–25.2]). Of the 170 pregnancies, 105 (62%) were unplanned. Unmet need for contraception was 50% higher in women initiating ART in the past year as compared to women on ART>1 year (prevalence ratio 1.5 [95% CI: 1.1–2.0]); by two years post-ART initiation, nearly one quarter of women had at least one unplanned pregnancy. Cumulative incidence of pregnancy was equally high among recent ART initiators and ART experienced participants: 23.9% [95% CI: 16.4–34.1], 15.9% [12.0–20.8], and 21.0% [16.8–26.1] for women on ART 0–1 yr, >1 yr–2 yrs, and >2 yrs respectively (log-rank, p = 0.54). Eight hormonal contraceptive failures were detected [IR: 4.4 [95% CI: 2.2–8.9], 7/8 among women using injectable methods. Overall 47% (80/170) of pregnancies were not carried to term.

**Conclusions:**

Rates of unintended pregnancies among women on ART are high, including women recently initiating ART with lower CD4 counts and higher viral loads. A substantial burden of pregnancy loss was observed. Integration of contraceptive services and counselling into ART care is necessary to reduce maternal and child health risks related to mistimed and unwanted pregnancies. Further research into injectable contraceptive failures on ART is warranted.

## Introduction

Women account for 60% of HIV-infections in Sub-Saharan Africa [1]. Contraception is critical to the health and empowerment of all women, particularly women infected with HIV. Preventing unintended pregnancies amongst women living with HIV is one of four components of the World Health Organization's (WHO) prevention of mother-to-child transmission (PMTCT) strategy and has been shown to be a cost-effective approach to HIV prevention [2]–[4]. Integration of HIV and reproductive health services can increase contraceptive uptake, but standard practice in most health systems is to provide separate services for HIV treatment and contraception [5], [6].

Individuals living with HIV vary in their desire for more children [7]–[9]. Lower fertility rates have been reported in HIV-positive women, however evidence suggests that pregnancy incidence increases after ART initiation [10]–[12]. For HIV infected women choosing to become pregnant, planning a conception at a time when her CD4 count is high and viral load low contributes to maternal wellbeing and to the prevention of HIV transmission to the unborn child [13], [14]. For these reasons there has been increasing recognition of the importance of appropriately-timed pregnancies.

While pregnancy rates are increasing among women on ART, it remains unclear to what extent this phenomenon is due to choice or due to an increase in unplanned pregnancies. Women may have experienced HIV-related sub-fertility in the past and may not anticipate an increased pregnancy risk upon ART initiation. Although women on ART are already linked into the health care system and therefore should have contraceptive access, limited integration of services and a focus on condoms alone as a method to prevent HIV transmission may result in inadequate contraceptive provision in this population and an increased risk for unintended pregnancies.

The objectives of this study are to determine the incidence of unplanned pregnancies in HIV-positive women on ART in South Africa, and to assess contraceptive use and associations with unplanned pregnancy in this population.

## Methods

### Ethics Statement

The study protocol was approved by the University of the Witwatersrand Human Research Ethics Committee, Johannesburg, South Africa. Written informed consent was obtained from all participants.

### Study design and recruitment

Data presented are from a prospective cohort study conducted in four government-run ART clinics in Johannesburg, South Africa. ART is provided free of charge by the government in South Africa based on immunological or clinical status. At the time of study enrolment, non-pregnant women were eligible for lifelong ART initiation with a CD4 count ≤200 cells/µl or WHO clinical stage 4 diagnosis [15].

Enrolment took place from August 2009 to January 2010 at two ART initiation sites and two ART down-referral sites where patients continue treatment once they are clinically stable. The down-referral sites are near to the study initiation sites and are comprised of demographically similar populations. All women of child-bearing age in clinic waiting rooms were informed by research assistants of the opportunity to participate in a study related to women's health and ART experience. Further screening and informed consent of interested individuals occurred in private rooms.

**Figure 1:**
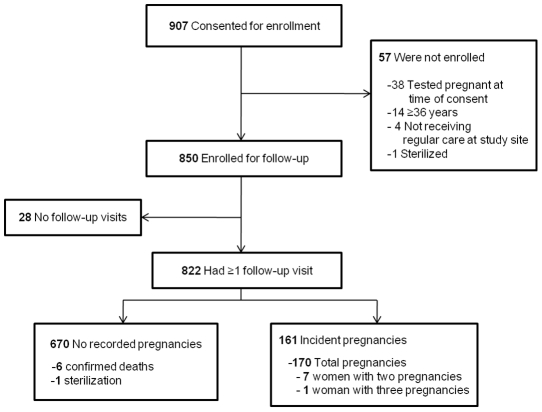
Study flow diagram of enrollment and follow-up.

Our sample size was determined to compare pregnancy incidence rates across ART regimens. The study targeted 850 participants for enrolment. For the present analysis, based on *a priori* expected cumulative pregnancy incidence of 15%, and an anticipated loss-to-follow-up of 15%, we would have >80% power to detect a 10% difference in pregnancy survival curves between women not trying to conceive with met and unmet contraceptive need. Women were eligible for participation if at enrolment they were: between 18–35 years, not pregnant, had not delivered within the three months prior, not breast-feeding, on ART or being initiated onto ART, sexually active in the past twelve months, and had not undergone prior tubal ligation, hysterectomy, bi-lateral oophorectomy, or otherwise been diagnosed permanently infertile. Those with a positive pregnancy test at time of recruitment were not eligible for study enrolment and were referred to antenatal care or other appropriate services. Non-pregnant women completed a baseline interview and were enrolled for study follow-up. Approximately 9,000 persons were receiving care at study sites, of which around 3,000 were women between the ages 18–35 years. Overall 907 women were consented to participate in the study. Of these women, 57 (6%) were excluded as they were determined to be ineligible and 850 women were enrolled (Figure 1).

### Data Collection and Follow-up

Baseline interviews were conducted in English, Zulu or Sotho by trained study staff in private clinic rooms. Interviews covered demographics, ART experience, obstetrics history, contraceptive use, fertility intentions, communication with providers and questions about partnership dynamics.

Women were seen by study staff during routine clinic visits over one-year of follow-up. The median time between visits was 1.8 months [interquartile range (IQR) 0.9–2.1]. Contraceptive use, current fertility intentions and pregnancy testing were assessed at each study visit. Participants were referred for antenatal care or other support services as indicated. Study follow-up for non-pregnant women was concluded at the first clinic visit after completing one year of follow-up. Pregnant women were followed through the duration of pregnancy to assess reproductive outcomes. Overall, 91% of women contributed two or more follow-up visits after their baseline visit.

Date of ART initiation was confirmed through clinical and pharmacy records. ART regimen information, CD4 cell count and viral load were collected longitudinally and confirmed through pharmacy and laboratory records. Patient files were reviewed to collect additional clinical and treatment history and to abstract any pregnancies or reproductive health outcomes during study follow-up that may have been missed.

### Variable Measures

Pregnancy was determined using urine-based tests administered by study staff (*One Step hCG Urine Pregnancy Test, Atlas Link Technology, Beijing*). Positive pregnancy tests were confirmed through repeat testing. Conception dates were assigned to two weeks following the last menstrual period (LMP) or if unknown, to 266 days before the due date determined by ultrasound and recorded in antenatal records.

Contraceptive use was self-reported. Women were considered to be using a reliable method of contraception at baseline if they reported using hormonal contraception (HC), consistent condom use, or both. Hormonal method use included depot-medroxyprogesterone (DMPA) and norethisterone enantate (NET-EN) injectable methods, combined oral contraceptives (COCs), and hormonal implants. There was only one participant using the intrauterine device (IUD) who for the purposes of this analysis has been grouped with HC users. Consistent condom use at baseline was defined as using a condom during the last sexual act and reporting condom use every time over the past twelve months.

This study considered unmet need for contraception to include all women in situations of risk for an unplanned pregnancy. Specifically, baseline unmet need for contraception included women at enrollment who were: (i) married or living with a partner, or had sexual intercourse in the past three months, and (ii) reported that they were not trying to conceive, and (iii) were not using a reliable form of contraception as defined above.

### Statistical analysis

Comparisons of medians and categorical variables were calculated using non-parametric k-sample tests of equality of medians and chi-squared tests respectively. Any comparisons between groups presented in the text use chi-squared tests unless otherwise noted.

Incidence rates were calculated for the first incident pregnancy per 100 person-years (PYs). Rates for planned and unplanned pregnancies included only time in the denominator during which women were at risk for planned pregnancies or unplanned pregnancies respectively.

Survival analysis was used to assess time-to-pregnancy and time-to-unplanned pregnancy by ART duration and contraceptive use. The origin for the survival analyses was time of study enrolment. Events were defined as the date of pregnancy. Persons were censored from the analysis if they were lost to follow-up, withdrew from the study, died or upon study completion. Kaplan-Meier (KM) curves were used to plot failure curves. Risk sets for the KM curves plotting the incidence of unplanned pregnancy include only periods of person-time contributed when women were not trying to conceive and were thus at risk for an unplanned pregnancy. Log-rank tests were used in the KM analyses to assess the equality of failure functions across groups. Exposures for fertility intentions, contraceptive use and time-on-ART are time-varying in the KM analyses.

Univariate and multivariate analyses to assess predictors of baseline unmet contraceptive need were calculated using robust poisson regression models to estimate prevalence ratios (PRs) and adjusted prevalence ratios (aPRs). Poisson regression was the preferred estimation method as the frequency of the outcome was greater than 10% [16]. Variables were included in the multivariate analysis based on prior hypotheses or demonstrated statistical significance using a two-sided, α≤0.10 threshold in the univariate analyses. Twenty-three (3%) of participants were missing income values. As described previously, multiple imputation with Rubin's pooling method was used in the regression analyses to restore missing income values [17], [18]. Data were analyzed using Stata version 11.1 (*StataCorp, College Station, Texas, USA*).

## Results

### Study population and follow-up

Of the 850 women enrolled, 822 (97%) had at least one additional follow-up visit between August 2009 and March 2011. Participants had a median of six post-enrolment follow-up visits and contributed a median of 12 months [IQR 9–13] of follow-up to the cohort.

Table 1 summarizes baseline characteristics of the cohort overall and by time-on-ART. The average participant was 30 years of age [IQR 27–33] and had one living child [IQR 1–2]. Time-on-ART at baseline in the cohort ranged from 0–7.4 years (median 1.1 [IQR 0.4–2.0]) and the baseline CD4 cell count ranged from 3–1451 cells/µl (median 320 [IQR 178–469]). Women differed substantially at enrolment in their demographic and clinical characteristics according to time-on-ART. Relationship and marital status, partner HIV-status, disclosure to a partner, and having a child with a current partner were characteristics that were comparable across groups (Table 1).

**Table 1:** Description of baseline cohort characteristics by months-on-antiretroviral therapy (ART), South Africa.

Characteristics at time of study enrollment	All participants (n = 850)	Recent ART Initiators	ART Experienced		p-value
		(0–12 mo)	(>12 mo–24 mo)	(>24 mo)	
		n = 399	n = 240	n = 211	
**Median (IQR)** a					
**Age, yrs**	30 (27–33)	30 (27–32)	30 (27–33)	32 (28–34)	0.008
**Monthly income, USD**	270 (149–473)	270 (135–405)	270 (162–473)	323 (189–541)	0.012
**Time on ART, months**	13 (5–24)	5 (2–8)	17 (14–20)	37 (30–48)	<0.001
**CD4 count at enrollment, cells/µl**	320 (178–469)	183 (124–301)	381 (284–515)	503 (366–650)	<0.001
**Most recent HIV-1 Viral load, log_10_ (copies/ml)** b	1.7 (1.7–1.8)	1.7 (1.7–2.8)	1.7 (1.7–1.7)	1.7 (1.7–1.7)	<0.001
**Number of living children**	1 (1–2)	1 (0–2)	1 (1–2)	1 (1–2)	0.013
**Relationship duration, yrs** c	4 (2–7)	3 (1–6)	5 (2–7)	4 (2–8)	0.065
**Time since last pregnancy, yrs** d	4 (2–8)	5 (2–8)	3 (1–7)	3 (2–7)	<0.002
**n (%)**					
**Employed**	510 (60)	222 (56)	145 (60)	143 (68)	0.014
**Education completed**					
None – grade 10	218 (26)	121 (30)	55 (23)	42 (20)	0.020
Grade 11–12	528 (62)	232 (58)	160 (67)	136 (64)	
Post-grad degree or certificate	104 (12)	46 (12)	136 (10)	33 (16)	
**In a relationship**	790 (93)	365 (91)	227 (95)	198 (94)	0.280
**Multiple current partners**	100 (12)	48 (12)	18 (8)	34 (16)	0.018
**Sexually active past 3 mos.**	758 (89)	345 (86)	221 (92)	192 (91)	0.053
**Married/Cohabitating**	378 (44)	185 (46)	102 (43)	91 (43)	0.574
**Disclosure to main partner** c	640 (81)	294 (81)	189 (83)	157 (79)	0.555
**Partner HIV status** c					
HIV-negative	200 (25)	93 (25)	54 (24)	53 (27)	0.881
HIV-positive	312 (40)	141 (39)	90 (39)	81 (41)	
Unknown HIV-status	278 (35)	131 (36)	83 (37)	64 (32)	
**≥1 living child with ** ***current*** ** partner** c	324 (41)	136 (37)	105 (46)	83 (42)	0.098
**Currently trying to conceive**	102 (12)	58 (15)	19 (8)	25 (12)	0.044
**Using hormonal contraception**	243 (29)	91 (23)	82 (34)	70 (33)	0.002
**Experienced violence in current or past relationship**	86 (10)	50 (13)	21 (9)	15 (7)	0.076
**Pregnant during or since HIV diagnosis**	380 (45)	123 (31)	129 (54)	128 (61)	<0.001
**Normal menstrual cycle during past 3 months**	554 (65)	273 (68)	141 (59)	140 (66)	0.042

a IQR = interquartile range;

b n = 834;

c Restricted to participants in a current relationship (n = 790);

d Restricted to participants with a prior pregnancy (n = 760).

### Incidence of pregnancy and time-on-ART

Over the course of follow-up, 170 new pregnancies were detected in 161 women over 745.2 PY at risk (incidence rate: 21.6/100 PY [95% CI 18.5–25.2]; Figure 1). Of these, 105 (62%) of the pregnancies were unplanned (incidence rate: 16.1/100 PY [95% CI 13.2–19.7; Table 2). Amongst all women who conceived, median-time to first pregnancy was 168 days [IQR 85–260]. Median-time to first unplanned pregnancy was 136 days [IQR 84–253].

**Table 2:** Incidence of pregnancy in the cohort of South African women, August 2009–March 2011.

	Time-at-risk	Events	Incidence Rate (per 100 PY)
	(Person Years [PY])		[95% Confidence Interval]
**Total pregnancies**	**745.2**	**161**	**21.6 [18.5–25.2]**
Unplanned pregnancies	602.1	97	16.1 [13.2–19.7]
Planned pregnancies	143.2	64	44.7 [35.0–57.1]

**Figure 2:**
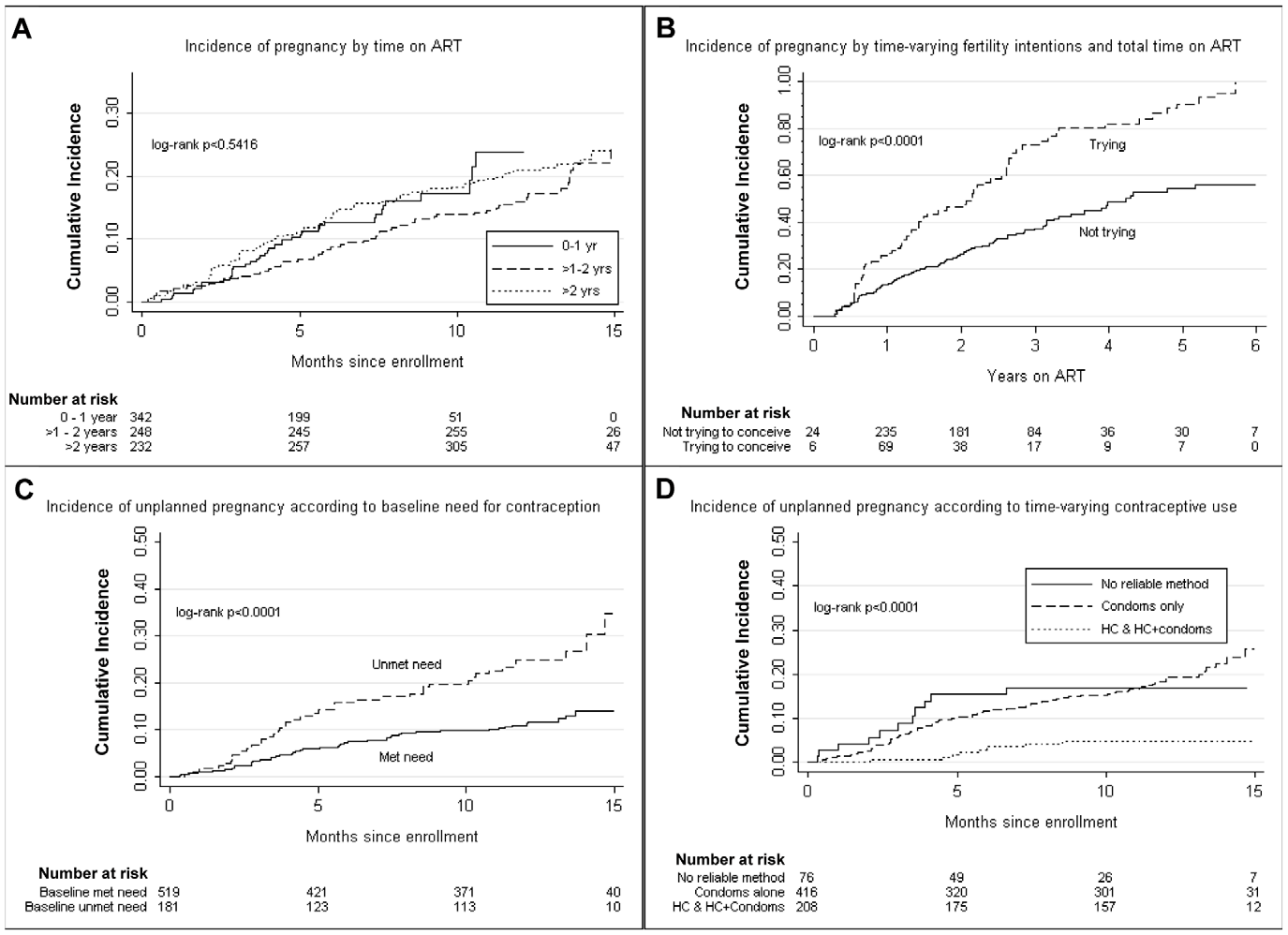
Kaplan-Meier plots of time-to-first pregnancy amongst women on antiretroviral therapy (ART), South Africa. Time-to-first pregnancy is depicted by: (A) total years-on-ART; (B) time-varying fertility intentions; (C) baseline need for contraception; and (D) time-varying contraceptive use during study follow-up. *HC = hormonal contraception*.

Figure 2 shows the estimated failure functions for time-to-first pregnancy by covariates of interest. There was no statistically significant difference in time-to-pregnancy across duration of ART; cumulative incidence of pregnancy after 12 months of follow-up for ART 0–1 year, >1 yr–2 years, and >2 years was 23.9% [95% CI 16.4–34.1], 15.9% [95% CI 12.0–20.8], and 21.0% [16.8–26.1] respectively (log rank, p = 0.54). Considering time-on-ART as the time metric rather than study time, by two years on ART, nearly one quarter of women had at least one unplanned pregnancy and by three years the number increased to more than one-third (Figure 2b). To assess the robustness of this finding, we repeated this analysis excluding those women who had a prior pregnancy while on ART (n = 105) and findings were similar (results not shown). There were no differences in pregnancy incidence by time-varying CD4 cell count categories, time-varying viral load, partners' baseline HIV-status, nor enrolment site (results not shown).

Outcomes to-date of the 105 unplanned pregnancies are 38 (36.2%) elective terminations of pregnancy, 19 (18.1%) spontaneous abortions, 1 (0.95%) ectopic pregnancy, 1 stillbirth (0.95%), 42 (40.0%) live births, and 4 (3.8%) on-going pregnancies.

### Contraceptive use and incidence of unplanned pregnancy

Figure 2c compares incidence of unplanned pregnancy according to baseline unmet need for contraception. These curves illustrate that women with a baseline unmet need for reliable contraception had a higher incidence of unplanned pregnancy during study follow-up as compared to women who reported use of a reliable contraceptive (log-rank p<0.01). When considering time-varying contraceptive use and incidence of unplanned pregnancy (Figure 2d), women reporting condom use alone to prevent pregnancy had similar cumulative incidence rates of unplanned pregnancies to women reporting no reliable method use; women on HC had significantly lower incidence rates.

Nine of 105 unplanned pregnancies (8.6%) were hormonal contraceptive failures. All contraceptive failures were confirmed through review of family planning records. One COC failure appears to have been due to poor compliance and is excluded from the subsequent calculations. The incidence rate of unplanned pregnancy on HCs was 4.4/100 PY [2.2–8.9], including one failure not related to compliance on a COC (5.8/100 PY [1.4–23.0]) and seven injectable failures (2 DMPA, 5 NET-EN) (4.2 [1.9–9.4]). Five of the seven injectable failures were estimated to have occurred during the final two weeks of the injection cycle. Regarding the ART regimens of women experiencing HC failures, seven women were on nevirapine-based ART and one failure was on an efavirenz-based regimen. At baseline 94% of women were on first-line ART including nevirapine (52%) or efavirenz (42%).

### Prevalence of contraceptive use and predictors of unmet need for contraception

Prevalence of baseline HC use in the cohort was 28.6% (243/850). Progestin-only injectables were the most commonly reported method (n = 192), followed by COCs (n = 46), implants (n = 4) and IUD (n = 1).

Amongst women sexually active in the past three months and not trying to conceive, 54% of women reported consistent condom use and 33% were using HC, including 15% dual method users. Twenty-eight percent of sexually active women had an unmet need for reliable contraception at baseline. In the multivariate analysis (Table 3), women initiating ART in the past year had a 50% higher prevalence of unmet need for contraception as compared to women on ART for greater than one year (aPR 1.5 [95% CI 1.1–2.0], p = 0.01). Having a CD4 cell count ≤200 was significantly associated with an unmet need for reliable contraception in comparison to other CD4 categories.

**Table 3:** Predictors of unmet need for contraception amongst sexually active women not trying to conceive (n = 675).

	Univariate PR^a^	p-value	Multivariate aPR^b^	p-value
	[95% CI]		[95% CI]	
**Age (per 5 years)**	0.78 [0.67–0.90]	0.001	0.90 [0.77–1.05]	0.169
**Employed**				
No	**REF**			
Yes	0.91 [0.71–1.17]	0.477		
**Education Completed**				
None - grade 10	**REF**			
Grade 11–12	0.89 [0.68–1.17]	0.415		
Post grad degree/certificate	0.91 [0.60–1.39]	0.660		
**Income Category, (USD/mo)**				
$0–200	**REF**		**REF**	
$201–400	0.81 [0.60–1.08]	0.151	0.85 [0.64–1.13]	0.262
>$400	0.69 [0.50–0.94]	0.019	0.74 [0.55–1.01]	0.058
**Married/Cohabitating**				
No	**REF**			
Yes	0.82 [0.64–1.05]	0.108		
**Number of living children**				
None	**REF**		**REF**	
One	0.82 [0.61–1.10]	0.190	0.91 [0.68–1.21]	0.497
Two or more	0.57 [0.41–0.79]	0.001	0.72 [0.51–1.01]	0.057
**Baseline CD4 category at enrollment, cells/µl**				
≤200	**REF**		**REF**	
201–350	0.48 [0.35–0.68]	<0.001	0.57 [0.41–0.80]	0.001
351–500	0.52 [0.37–0.72]	<0.001	0.67 [0.47–0.95]	0.026
>500	0.51 [0.36–0.72]	<0.001	0.74 [0.49–1.11]	0.147
**Time since ART initiation**				
>1 yr (ART experienced)	**REF**		**REF**	
≤1 yr (recent initiator)	1.88 [1.47–2.42]	<0.001	1.47 [1.08–1.99]	0.013
**Prior exposure to intimate partner violence**				
No	**REF**		**REF**	
Yes	1.37 [0.98–1.92]	0.069	1.35 [0.98–1.88]	0.069
**Concurrent partners**				
No	**REF**		**REF**	
Yes	1.54 [1.14–2.07]	0.004	1.41 [1.06–1.87]	0.017

a,b Prevalence Ratios (PRs) and adjusted Prevalence Ratios (aPRs) estimated using robust Poisson regression.

### Communication with providers and barriers to using HC

Fewer than half (48%) of women reported that an HIV provider had discussed hormonal contraception with them. Of the 243 women using HC, 82 (34%) reported experiencing problems with their method. Over three-quarters of all problems (79%) were related to menstrual bleeding, including amenorrhea (n = 31), heavy bleeding (n = 14) or other irregular bleeding (n = 20). The majority (61%) of women experiencing problems with their HC method had not discussed these with any provider. Furthermore, in this setting, women see different providers for their HIV treatment and contraceptive care and over half of women (55%) had not informed their contraceptive care providers of their HIV-status.

Amongst those not currently trying to conceive and not using HC, the most commonly reported reasons for not using HC were: already using condoms (n = 184, 36%), irregular bleeding (n = 61, 12%), and general side effects (n = 53, 10%).

## Discussion

We have documented high rates of unplanned pregnancies after ART initiation in South Africa; these high rates are at least partially driven by a high unmet need for contraception during the first year of ART. These findings underscore the need to counsel women about pregnancy risk at time-of-ART initiation and consistently thereafter. Moreover, these results provide preliminary data on the magnitude of health risks that could in part be averted through the integration of contraception services into ART treatment services. Preventing unwanted pregnancies can reduce maternal morbidity risks related to unsafe abortion and minimize any negative impacts of pregnancy on health, especially among women with lower CD4 cell counts. Prevention of unintended pregnancies is also critical to reduce MTCT risks, particularly in the period soon after ART initiation before HIV viral suppression has been achieved.

The incidence rate of 21.6 pregnancies/100 PY is twice as high as that recently reported amongst HIV uninfected women in a multi-site HIV Prevention Trials Network study (HPTN 035), however HC use was also two times higher in HPTN 035 [19]. When comparing our study's pregnancy incidence rates to those of other studies amongst HIV infected women, the overall incidence is similar to a recent report from Uganda, but higher than several other studies [11], [12], [20], [21]. As many studies rely on less frequent pregnancy testing and/or self-report, it is not surprising that the pregnancy rates reported in this study are higher. In this setting 105/170 (62%) pregnancies were unplanned and of these, 57 (54%) were not carried to term; had we relied on self-report and not tested regularly for pregnancy, many of these pregnancies would likely have remained undetected. The high burden of termination of pregnancy, 26% of all pregnancies and 38% of unplanned pregnancies, further highlights the consequences of unmet need for contraception in this population.

We might have expected women recently initiating ART to have had lower rates of pregnancy during this period of immunological reconstitution. Pregnancy incidence rates, however, were highest during the first year after ART initiation and statistically comparable to those of more ART-experienced women. Unmet contraceptive need was also greatest in the year following ART initiation. It is particularly concerning that by two years on ART, 25% of women had at least one unplanned pregnancy and this number increased to 35% by three years on ART.

While family planning is not a routine concern of HIV staff during ART initiation, these results suggest that it should be an immediate priority for providers and discussed regardless of CD4 cell count or perceived risk of pregnancy. Sexual activity is likely to increase after ART initiation as health improves, and in the absence of counselling and family planning, high rates of unplanned pregnancies will occur even amongst women with low CD4 cell counts. Counselling should emphasize that pregnancy risk is similar in women on ART as compared to HIV-uninfected women, and that women may be at risk for pregnancy while amenorrheic [22]. Side effects, including menstrual irregularities, may be a cause for greater concern amongst HIV-infected women and should be addressed in counselling. Down-referral ART sites offer HC onsite, though in separate rooms by separate providers. We did not see a difference in unplanned pregnancies between sites however it is likely that rates at primary health care down-referral sites would be even higher if contraception were unavailable on-site. Further integration of HC services into ART care may improve method uptake, streamline counselling messages and decrease clinic visit times.

At baseline only one-third of sexually active women not intending to conceive were using HC. Our findings reinforce results from other studies, however, that condom use alone does not substantially decrease pregnancy risk and is generally not a reliable method of contraception for women on ART [11], [23]. Limited uptake of HC may be in part due to poor patient-provider communication. HIV providers may be wary of promoting HC based on suspicions of condom migration, mixed evidence around the impact of HC on women's health, due to concerns over potential drug interactions resulting in reduced HC efficacy, or due to inadequate training in HC service provision [24]–[27]. Recent findings that HCs may increase HIV transmission from women not using ART to uninfected male partners are cause for further concern and will need to be considered alongside maternal and infant risks associated with unintended pregnancies [28].

We reported eight conceptions in women using HC that were not failures attributed to compliance. Seven of the failures were in women on NVP-based regimens. Further attention to this issue is warranted as the observed failure rate of 4.2/100 PY on injectable methods is much higher than 0.3/100 PY, the estimated failure rate for perfect use in non-ART populations [29]. As five out of the seven injectable failures occurred towards the end of the injection cycle, providers may need to consider reducing the time between injections for women on ART. IUDs have been shown to be safe for HIV-positive women and could be promoted as an alternative to avoid possible drug interactions [24].

This study has some limitations. Hormonal contraception was self-reported and while condom use was recorded throughout the study, consistency of condom use and frequency of sex were only assessed at baseline and during the final interview. Nevertheless, the incidence analysis using baseline unmet need provides similar inferences to the analysis using longitudinal contraception use. Other variables including changes in partnership status and last menstrual period were not collected at each visit, but would have been informative as they may reflect changes in pregnancy risk. Exit interviews indicated that 91% of women were in relationships at end of follow-up as compared to 93% at baseline. Although some women did experience changes in relationships, partnership gap times cannot be determined in our data. Another concern may be that there was a selection bias into the study such that women more likely to conceive were more likely to participate. We intentionally limited the study population to sexually active women aged 18–35, as these are the women most at-risk for pregnancy and the population to which these findings should be targeted in a clinical setting. While it is possible that women desiring more children were more likely to participate, study recruitment messages expressed a general interest in women and reproductive health. Furthermore, the high rate of pregnancy termination observed suggests that the unplanned pregnancies genuinely were unwanted.

As all women in our population are on ART, we cannot attribute the high rates of unplanned pregnancy to ART initiation. The findings from this study do suggest however, that reproductive health-related risks are neglected around the time-of-ART initiation and pregnancy risk is high in the year following ART initiation. In order to decrease unintended pregnancies and associated health risks in ART populations, HIV providers should be trained in HC provision. ART providers have experience in counselling patients related to adherence and side effects, and are a point of care women routinely access. While more research is necessary around contraceptive failures on ART, the risk of pregnancy on HC remains significantly lower than in women using condoms alone. Contraception, including HC and IUDs alongside condoms, should be routinely offered to women on ART. Information regarding benefits and side effects of different methods should be provided in order to ensure informed choices are supported and unnecessary method discontinuation avoided.
